# Duration of protective immunity after a single vaccination with a live attenuated bivalent bluetongue vaccine

**DOI:** 10.1007/s11259-015-9643-4

**Published:** 2015-08-18

**Authors:** Kuandyk Zhugunissov, Zakir Yershebulov, Kainar Barakbayev, Yerbol Bulatov, Dmitriy Taranov, Zhanat Amanova, Yergali Abduraimov

**Affiliations:** Research Institute for Biological Safety Problems, Gvardeiskiy, Kordai Raion, 080409 Zhambyl Oblast Republic of Kazakhstan

**Keywords:** Bluetongue, Vaccine, Safety, Immunogenicity

## Abstract

The prevention of bluetongue is typically achieved with mono- or polyvalent modified- live-attenuated virus (MLV) vaccines. MLV vaccines typically elicit a strong antibody response that correlates directly with their ability to replicate in the vaccinated animal. They are inexpensive, stimulate protective immunity after a single inoculation, and have been proven effective in preventing clinical bluetongue disease. In this study, we evaluated the safety, immunogenicity, and efficacy of a bluetongue vaccine against *Bluetongue virus* serotypes 4 and 16 in sheep. All the animals remained clinically healthy during the observation period. The vaccinated animals showed no clinical signs except fever (>40.8 °C) for 2–4 days. Rapid seroconversion was observed in the sheep, with the accumulation of high antibody titers in the vaccinated animals. No animal became ill after the challenge, indicating that effective protection was achieved. Therefore, this vaccine, prepared from attenuated *bluetongue virus* strains, is safe, immunogenic, and efficacious.

## Introduction

Bluetongue is a viral disease transmitted by *Culicoides* spp. Infection is characterized by fever, congestion, edema, hemorrhage, hyperemia and ulceration of the oral mucosa, coronitis, and lameness in domestic and wild ruminants (Roy 2002). *Bluetongue virus* (BTV) belongs to the family *Reoviridae* and is the type species of the genus *Orbivirus*, which also includes the *epizootic hemorrhagic disease virus* of deer and *African horse sickness virus*. BTV has a high level of antigenic variation, with 27 serotypes recognized worldwide (Jenckel et al. 2015).

Bluetongue was first described in Africa at the beginning of the 20th century and was considered exotic in Europe before1998. Since then, at least six serotypes (BTV-1, -2, -4, -8, -9, and -16) have invaded the European mainland on different occasions (Bréard et al. 2011), with a recent epizootic outbreak. The disease is mainly spread through trading of breeding animals from bluetongue-enzootic countries (Murueva 2011). According to data from the Ministry of Agriculture of the Republic of Kazakhstan on beef imports, over 50,000 head of cattle were delivered to Kazakhstan from the United States, Czech Republic, Canada, Russia, Ukraine, France, Australia, Ireland, Austria, and Germany in 2011–2014 (http://kapital.kz/economic/30953/rk-ogranichit-import-krupnogo-rogatogo-skota.html). Importation creates a risk of the emergence of bluetongue because many of these countries have enzootic bluetongue infections (OIE, Terrestrial Animal Health Code 2011). Over the past 15 years, Kazakhstan has conducted no serosurveys to monitor BTV infection, although neighboring countries contain seropositive animals (Zhugunisov et al. 2009; Vishnyakov et al. 1995) and have isolated different serotypes of BTV (Avci et al. 2014; Yang et al. 2011; Yang et al. 2012). Moreover, Lundervold et al. (2004) found BTV circulating in Kazakhstan among ruminants more than 10 years ago. Therefore, Kazakhstan is at an unknown, but significant, risk of bluetongue.

Bluetongue is typically prevented with mono- and polyvalent modified live virus (MLV) vaccines, inactivated (killed) virus vaccines, virus-like particles produced from recombinant baculoviruses, or recombinant *Vaccinia*- or -*Canarypox* virus-vectored vaccines (Savini et al. 2008; Noad and Roy 2009). MLV vaccines typically elicit a strong antibody response, which correlates directly with their ability to replicate in the vaccinated animal. The vaccines are inexpensive, stimulate protective immunity after a single inoculation, and have been proven effective in preventing clinical disease (Savini et al. 2008; Patta et al. 2004).

Given the recent spread of bluetongue to areas around Kazakhstan and worldwide, there is significant interest in developing an efficacious and safe vaccine against BTV serotypes 4 and 16, because these are most prevalent in the areas surrounding Kazakhstan. No information is currently available on the duration of the protective properties of live-attenuated vaccines against bluetongue. Therefore, in this study, we undertook developing and testing of an attenuated bivalent vaccine against BTV, and examining the protection it confers after a single immunization.

## Materials and methods

### Viral strains

We used the BTV strains Khuroson-40/13/4 (BTV-4) and RT/RIBSP40/13/16 (BTV-16) (Sametova et al. 2013), which were obtained in lyophilized form from the laboratory of the “Collection of Microorganisms” at the Research Institute for Biological Safety Problems (RIBSP) and refreshed in Vero cells. Both strains were isolated individually by serial passages in chicken embryos (to passage 40), then in Vero cell culture (passage 10). To determine the reversion of the attenuated viruses, the viral material was passaged in mice (1–3 days of age) and sheep (6–12 months of age). The sheep and mice remained alive, with no clinical signs of infection for 30 days. Both animal models, which are commonly used to evaluate the attenuation of BTV, are sufficient to test the attenuated strains (Franchi et al. 2008). By this work, the results of the study are presented in detail in a previously published paper (Sametova et al. 2013). We have also obtained patents for strains Khuroson-40/13/4 (patent #2013/1344.1) and RT/RIBSP40/13/16 (patent #2013/1345.1) (https://gosreestr.kazpatent.kz/ru/Search%20Patent). The viral material was titrated in Vero cell cultures, and the viral titers were expressed in log_10_ tissue culture infective doses (TCID)_50_/mL, calculated with the method of Reed and Muench (1938).

### Animals and bioethics

A total of 288 3–6-month-old female Kazakh fat-tailed sheep were used in this study. The sheep for the experiments were kept in quarantine for 1 month holding thermometry, after a clinical examination and blood serum test for the presence of specific antibodies, with a competitive enzyme-linked immunosorbent assay (cELISA; ID-Screen Bluetongue Early detection ELISA, ID-Vet, Montpellier, France). All the sheep were healthy and seronegative for BTV 3 days before the first vaccination. The animals were randomly allocated to the vaccinated and unvaccinated groups. Each group was kept in a separate room and had free access to water and feed throughout the experiment. This study was performed in compliance with national and international laws and guidelines on animal handling, and the experimental protocol was approved by the Committee on the Ethics of Animal Experiments of the RIBSP of the Science Committee of the Ministry of Education and Science of the Republic of Kazakhstan (permit number: 0114/100).

### Preparation of the bivalent BTV vaccine

Each viral suspension (Khuroson-40/13/4 and RT/RIBSP40/13/16 vaccine strains) was clarified by centrifugation at 3000×*g* for 30 min. Viral suspension was then combined with a stabilizing medium (at a final concentration of 3 % peptone [Sigma–Aldrich, St. Louis, MO, USA] and 2 % lactose [Sigma–Aldrich]) in a ratio of 1:1. A total of 200,000 units of penicillin, 200 mg of streptomycin, and 5000 units of nystatin were added to the suspension, the volume was expanded to 1 L, and the solution was refrigerated at 4 °C for 10–12 h. The liquid was then divided into aliquots in 1 mL ampoules and lyophilized for storage.

### Vaccine safety

The safety ofvaccine was tested by injecting it subcutaneously to nine sheep at a dose of 10^6^ TCID_50_/mL. The paired control group was administered 1 mL of subcutaneous phosphate-buffered saline (PBS). After vaccination, the body temperatures of the sheep were checked daily and their clinical signs were monitored for 14 days. The animals that showed severe clinical signs (loss of more than 20 % body weight, frequent hunching, severe conjunctivitis, or any condition that prevented food or water intake) were euthanized.

### Testing for reversion of the vaccine to wild type

We used 30 seronegative sheep to test the seroconversion of the vaccine. The sheep were divided into 10 groups of three animals. The first group was administered the vaccine intravenously at a dose of 10^4^ TCID_50_/mL. To detect viremia 7–8 days after viral inoculation, blood samples were collected from the febrile animals in ethylenediaminetetraacetic acid. The blood samples were pooled and titrated in Vero cell cultures. The inoculum (10-mL) was administered to the second group of three seronegative sheep. Further testing was performed similarly with all 10 passages in three replicates each. The animals were observed as described above, for approximately 3 weeks, to evaluate their health status and viremia.

### Vaccine administration

Two hundred forty female sheep were inoculated subcutaneously with a single dose (10^4^ TCID_50/_mL) of vaccine. During the vaccination phase, the sheep were housed indoors in pens and their health status monitored (rectal temperature and clinical signs) for 1 year. Blood samples were taken by jugular venipuncture at regular intervals (days 7, 14, 21, 28, 90, 180, 270, and 360 post-vaccination). The sera were tested for antibody titers with the serum neutralization test (SNT) and ELISAs.

### Challenge study

Virulent strains BTV4 and BTV16 (5.5 LD_50_/mL for BTV4 and BTV16) were used to challenge the sheep. These BTV strains were isolated from sheep in the Republic of Tajikistan during monitoring research by the employees of the RIBSP in 2007 (Abduraimov et al. 2009). Both BTV types were plaque purified three times in Vero cells and their type specificity was verified with a microneutralization test. The strains had been passaged three times in sheep. The challenge material consisted of blood samples collected from BTV4- and BTV16-infected animals, which were lyophilized and submitted to the RIBSP collection of microorganisms.We used three vials of control BTV4 and BTV16 strains for the challenge. All the vials were opened vials with dried blood, dissolved in PBS, and pooled, separately. The vaccinated sheep (described above) were intravenously administered 10 mL of homologous virulent strains (5.5 log_10_ ELD_50_/ml for BTV4 and BTV16). The animals were observed for 30 days with daily measurements of body temperature, and were evaluated for clinical signs with a scoring system (Table 1). Vaccine immunogenicity was evaluated by comparing the reactions of the vaccinated and unvaccinated sheep to infectious challenge. The control sheep should score at least 10 points after infection. A difference between the vaccinated and unvaccinated sheep was considered insignificant if the average difference in the scores was 0–7 points, weak if the difference was 7–12 points, moderate if the difference was 12–16 points, and pronounced if the difference was >16 points (Sergeev et al. 1981).

**Table 1:** Assessment of clinical signs of bluetongue in sheep

Category	Clinical sign	Score
Fever (temperature 40.1 °C and higher)	1–3 days with a peak of up to 41 °C	1
	1–3 days with a peak greater than 41 °C	2
	4 or more days with a peak of up to 41 °C	3
	4 or more days with a peak greater than 41 °C	4
Mucous membranes (eyes, nose, mouth)	Mild hyperemia	1
	Severe hyperemia	2
	Cyanosis	3
Cervical swelling	Mild swelling of the eyelids or lips	1
	Obvious swelling of the eyelids or lips	3
	Swollen muzzle	4
Erosion or hemorrhage	Erosions in the oral and nasal cavities or hemorrhages on the nares or nasal planum	3
Keratitis	Focal unilateral keratitis	2
	Diffuse unilateral or multifocal bilateral keratitis	3
	Diffuse bilateral keratitis	4
Attitude	Mildly depressed	1
	Moderately depressed	2
	Severely depressed with anorexia	3
Muscle weakness	Mild	1
	Pronounced, with or without lameness	2
Lacrimation		1
Nasal secretions	Mucous	1
	Purulent	2
Sialorrhea	Sialorrhea or foaming at the mouth	1
Diarrhea		1
Body condition	Decreased body condition	1
	Emaciation	2
Maximum score		30

### Group-specific ELISAs

The BTV-specific antibodies in the sheep sera were detected with a competitive ELISA (cELISA, ID-Screen Bluetongue Early detection ELISA, ID-Vet, Montpellier, France) directed against VP7, according to the manufacturer’s instructions. For this study, a threshold value of 40 % negativity (PN) was used to discriminate between positive (PN < 40) and negative (PN ≥ 40) BTV ELISA results.

### Detection of neutralizing antibody response in the sera of vaccinated animals

SNT was performed according to the method of Haig and MaRAA (1956) using the BTV-4 and BTV-16 reference strains and serotype-specific BTV-4 and BTV-16-positive control antisera. Briefly, the sheep sera were diluted (1:2 to 1:128) and titrated against 100 TCID_50_ of the BTV-4 vaccine strain. BTV16 was analyzed separately, in a similar way. The plates were incubated for 1 h at 37 °C, and then maintained at 4 °C overnight. The following day, 50 μL of a Vero cell suspension (2 × 10^5^ cells/mL) was added to each well, and the plates were incubated for 4–7 days at 37 °C. The wells were then scored for a cytopathic effect. The neutralization titer was determined as the dilution of serum giving a 50 % neutralization end point.

### Statistical analysis

All statistical analyses were performed with GraphPad Prism® version 6.0. Two-way analysis of variance was used to compare the rectal temperatures, clinical scores, and serology of the groups, with *P* ≤ 0.05 considered statistically significant.

## Results

### Safety of test series of the bivalent live cultural vaccine against BTV

Other than a relatively mild fever (40.3–40.8 °C) for 2–4 days and swelling at the site of vaccination which resolved within 2–3 days, the animals developed no clinical signs after vaccination (Table 2). These results indicate that this vaccine is generally safe for sheep.

**Table 2:** Vaccine safety

Inoculum	Animal number	Viral dose (TCID_50_/mL)/volume	Clinical signs or reaction of animals							
			Swelling	Fever	Stomatitis	Diarrhea	Conjunctivitis	Nasal secretions	Sialorrhea	Depletion
Live attenuated bivalent vaccine	134	1 × 10^6^/1.0	+	+	−	−	−	−	−	−
	182	1 × 10^6^/1.0	+	−	−	−	−	−	−	−
	163	1 × 10^6^/1.0	+	+	−	−	−	−	−	−
	132	1 × 10^6^/1.0	+	+	−	−	−	−	−	−
	336	1 × 10^6^/1.0	+	−	−	−	−	−	−	−
	181	1 × 10^6^/1.0	+	+	−	−	−	−	−	−
	178	1 × 10^6^/1.0	+	+	−	−	−	−	−	−
	333	1 × 10^6^/1.0	+	−	−	−	−	−	−	−
	192	1 × 10^6^/1.0	+	+	−	−	−	−	−	−
PBS (control)	398	0/1.0	+	−	−	−	−	−	−	−
	353	0/1.0	+	−	−	−	−	−	−	−

### Reversion to virulence

**Fig. 1:**
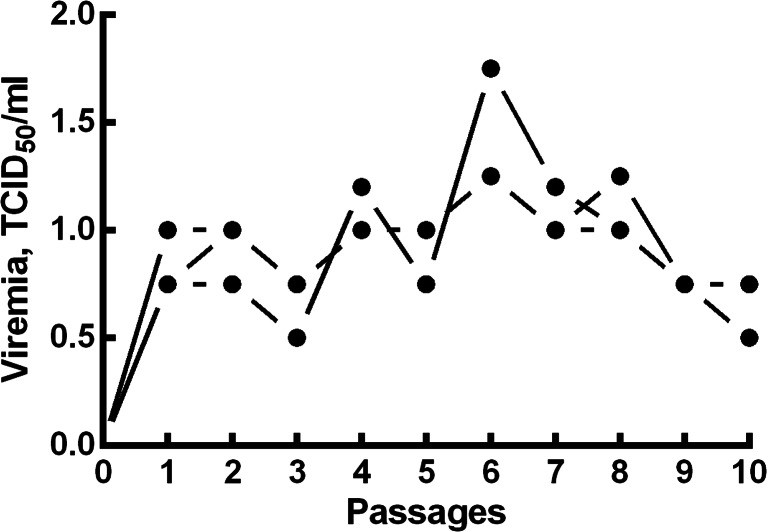
Viral titers during passage. We used 30 seronegative sheep in the experiment. The sheep were divided into 10 groups of three animals. The first group was administered the vaccine intravenously at a dose of 10^4^ TCID_50_/mL. To determine the level of viremia 7–8 days after viral inoculation, blood samples were collected from the febrile animals in ethylenediaminetetraacetic acid and titrated in Vero cell culture. The inoculum (10-mL) was administered to the second group of three sheep. Further testing was performed similarly in triplicate after each of the 10 passages

Both strains induced viremia in susceptible animals (Fig. 1). During the passages in sheep, the BTV titer was 1.16 ± 0.16 log_10_ TCID_50_/mL at the first passage, decreasing to 0.83 ± 0.08 and 0.92 ± 0.08 log_10_ TCID_50_/mL with subsequent passages (*P* ≤ 0.001) (10 total).

The attenuated virus showed no pathogenic properties after its intravenous administration to sheep, and all the animals remained healthy during the observation period (30 days).

### Neutralizing antibody titers

**Fig. 2:**
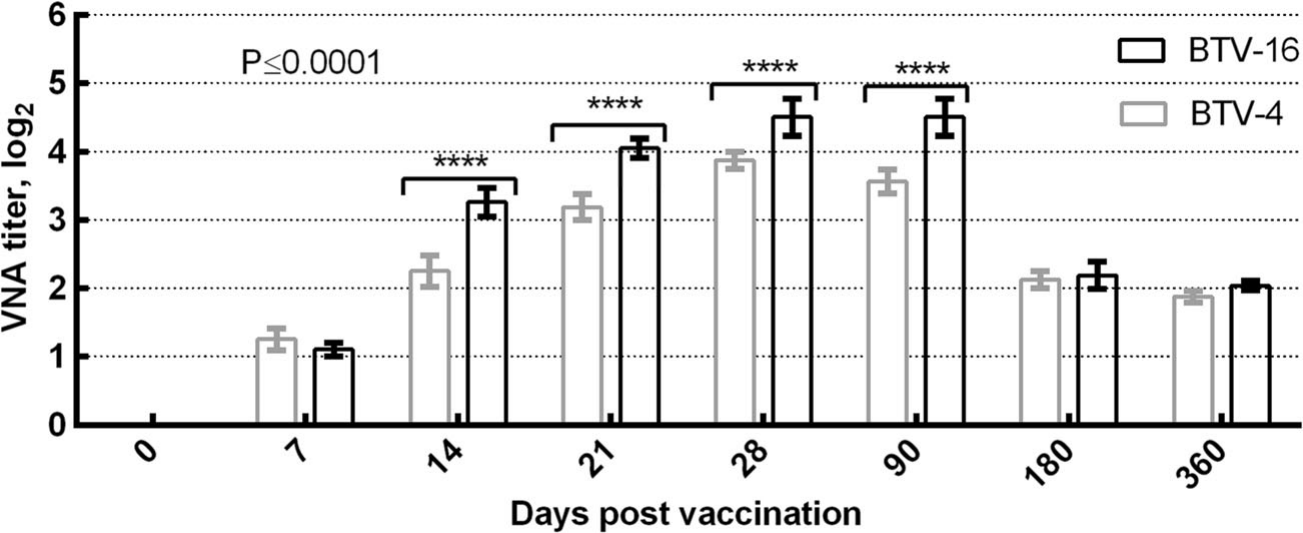
Dynamics of neutralizing antibodies in the vaccinated sheep. Data are means ± standard errors; ****Р < 0.0001. Neutralizing antibody titers were calculated with the Reed–Muench method and are expressed as log_2_ values

One week after vaccination, all the sheep had detectable levels of neutralizing antibodies, measured with a SNT, with mean titers (log_10_) ranging from 1.1 (for BTV-4) to 1.25 (for BTV-16) at 7 days after-vaccination (Fig. 2). Four weeks after vaccination, all the vaccinated animals had even higher levels of neutralizing antibodies: 4.0–4.8 log_2_ at 28 days and 1.8–2.0 log_2_ at 360 days (Fig. 2).

The levels of NA in the sera of the immunized sheep differed significantly for both serotypes from day 7 to day 360 (*P* ≤ 0.0001).

### Group-specific antibody titers

**Fig. 3:**
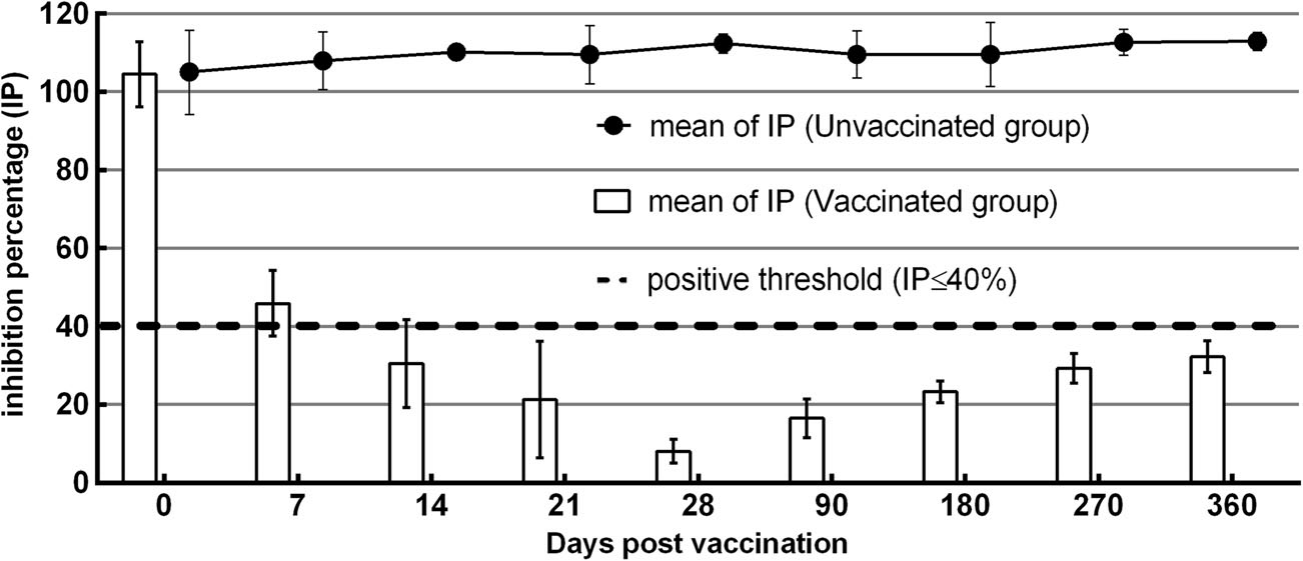
Evolution of mean percentage inhibition s (with standard deviation) in each group of vaccinated and control sheep during the experiment after a single vaccination. (anti-VP7 antibody ELISA)

A graphical summary of the percentage inhibition (IP) results are given for each group in Fig. 3. All sheep were seronegative before vaccination and the control sheep remained seronegative in all assays until challenge. After vaccination, the percentage inhibition decreased dramatically. The optical densities of specific antibodies decreased throughout the duration of the study, from IP < 8 ± 3.07 at day 28, to IP < 16.5 ± 4.95 at day 90, and IP < 46.25 ± 6.40 at day 360.

### Clinical protection following challenge

**Fig. 4:**
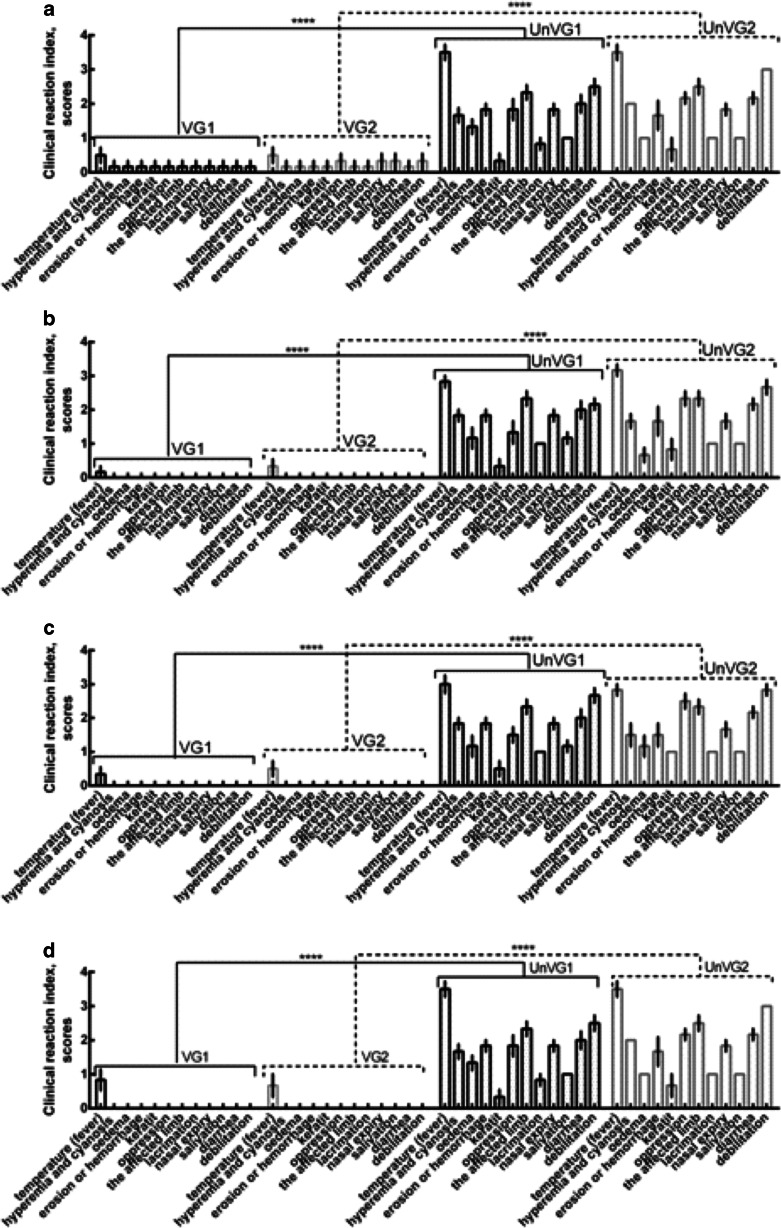
Assessment of the clinical signs in immunized sheep after infection with virulent BTV. **a** Challenge 7 days after vaccination. **b** Challenge 90 days after vaccination. **c** Challenge 270 days after vaccination. **d** Challenge 360 days after vaccination. *VG1* vaccinated group infected with virulent BTV-4. (*n* = 30) *VG2* vaccinated group infected with virulent BTV-16. (*n* = 30). *UnVG1* unvaccinated group infected with virulent BTV-4. (*n* = 4). *UnVG2* unvaccinated group infected with virulent BTV-16. (*n* = 4). Data are means ± standard errors; ****Р < 0.0001

Seven days after immunization, the animals developed protective immunity against BTV (Fig. 4a). However, the vaccinated animals were insufficiently protected from challenge because they developed clinical signs of bluetongue, with an average score of 10 points. The unvaccinated control animals also developed clinical signs, with an average score of 20.8 points, so the difference in the mean scores was 10.8 points. At 14, 90, and 270 days after immunization, strong protective responses developed against BTV-4 and BTV-16 (Fig. 4b, c). The vaccinated animals showed a slight increase in body temperature after challenge, whereas the unvaccinated animals developed typical clinical signs of the disease. At 360 days after immunization, one vaccinated sheep showed an increase in body temperature of 41.5 °C for 2 days after challenge, which then returned to normal (Fig. 4d). The vaccinated animals had an average clinical score of one point after challenge, whereas the unvaccinated animals had an average score of 27 points, indicating the high protective activity of the test vaccine.

## Discussion

Infection with BTV is common in a broad band across the world, which until recently stretched from latitude ~35°S to 40–50°N. Since the 1990s, the range of BTV has extended considerably north of the 40th and even the 50th parallels in some parts of the world (Maan et al. 2007; Coetzee et al. 2012).

All attenuated BTV strains have pronounced reactogenic properties. These properties are characterized by the appearance in the vaccinated animal of a quite moderate to severe reaction temperature, between 6 and 10 days after vaccination. The response to the vaccine is followed by viremia. These phenomena are regarded as positive signs of infection, indicating the development of an immune response (Kercher et al. 1957).

One of the most important steps when preparing a live-attenuated vaccine is the assessment of its level of attenuation in the target animals (sheep and newborn mice). These models, which are used to evaluate the attenuation of BTV, are sufficient to test the attenuated strains (Franchi et al. 2008). Therefore, we determined the levels of attenuation of the BTV strains with 10 serial passages in sheep. We evaluated the reversion of the two vaccine strains by passaging them 10 times through susceptible sheep, and thus showed that the pathogenicity of these attenuated strains was not restored. However, these strains replicated in the sheep and could be isolated from the blood of the animals 2–12 days after vaccination.

The MLV vaccine administered at doses not exceeding 2.0 log_10_ TCID_50_/mL, produced long-lasting viremia in the sheep, whereas virulent strains accumulated in the blood at levels up to 5.50 log_10_ TCID_50_/mL. Although this viremia makes the MLV more immunogenic, because it continues to producing an immune response long after the initial injection, the possibility of reversion to virulence cannot be ruled out given the limitations of the present study. In particular, previous research has shown that the virus can revert to virulence after infecting its natural vectors (Batten et al. 2008). In this case, the viremia produced by the vaccine strain persisted longer than that induced by the pathogenic strains. Vaccination with MLV and inactivated viruses produces neutralizing and group-specific antibodies (Oura et al. 2009; Hamers et al. 2009) protecting the vaccinated animal against challenge. Live-attenuated vaccines usually elicit a strong immune response, with the development of antibodies, and are usually efficacious after a single dose (Patta et al. 2004; Dungu et al. 2008).

Neutralizing antibodies have been shown to be an essential component of the protective immune response against BTV (Huismans et al. 1987; Roy et al. 1990) and similar titers, (1.5–2.5 log_2_) have been observed after vaccination with killed commercial vaccines in ruminants, which were ultimately protected from BTV-8 challenge (Bréard et al. 2011). Our experimental data are consistent with those of other researchers. Therefore, we have established that 7 days after vaccination with the attenuated vaccine, virus-neutralizing antibodies to BTV are present in the sera at a titers of 1.0 log_2_. This antibody titer is protective against the homologous virulent strain of BTV. Although further research isrequired, particularly regarding the safety of the vaccine in pregnant sheep, this bivalent vaccine has been shown to be safe and efficacious, and will be a valuable tool in preventing the spread of bluetongue to Kazakhstan.

## Conclusions

In conclusion, in an efficacy study, a single dose of an attenuated bivalent vaccine directed against BTV-4 and BTV-16 provided solid clinical protection against experimental challenge for 12 months. There was a strong anamnestic response in the vaccinated sheep and a good correlation between the neutralizing antibodies at the time of challenge and the protection afforded against both viral replication and clinical disease.
